# Computed tomographic findings of tuberous sclerosis with pulmonary lymphangioleiomyomatosis and renal angiomyolipomas: a case report

**DOI:** 10.4076/1757-1626-2-9238

**Published:** 2009-09-11

**Authors:** Flavia Gavinho Vianna, Edson Marchiori, Glaucia Zanetti, Claudia Mauro Mano, Branca Sarcinelli-Luz, Juliana Franca Carvalho, Carla Assed, Isabella Guedes Santos, Alair Augusto Santos, Alberto Domingues Vianna

**Affiliations:** 1 Department of Radiology, Faculty of Medicine, Fluminense Federal University, CEP 25685.120, Petropolis Rio de Janeiro, Brazil

## Abstract

The authors describe a case of a 31-year-old female with tuberous sclerosis, a genetic, rare, variably expressed disease. Clinical symptoms were chest pain, and progressive dyspnea. Computed tomography scan of the chest showed bilateral, diffuse, small thin-walled cysts scattered throughout the lungs characteristic for pulmonary lymphangioleiomyomatosis. Computed tomography scan of the abdomen revealed enlarged, heterogeneous kidneys, with low density tumors corresponding to angiomyolipomas. Pulmonary lymphangioleiomyomatosis and bilateral renal angiomyolipomas are some presentations of tuberous sclerosis and the coexistence of both conditions may cause devastating morbidity and mortality.

## Introduction

Tuberous sclerosis (TS) is an autosomal dominant disorder characterized by the formation of hamartomatous lesions in multiple organs, with a birth incidence of around one in 10,000 [1]. However, with more sensitive screening the prevalence may be as high as one in 6,000 [2,3]. The disease results from mutations in one of two genes, *TSC1* (encoding hamartin) or *TSC2* (encoding tuberin), which have an important role in the regulation of cell proliferation and differentiation [4]. Facial angiofibromas, renal angiomyolipomas, and pulmonary lymphangiomyomatosis (LAM) are some of the major features of this disease [4]. Diagnosis is usually established on the basis of physical examination, radiological findings or both, and the presentation of the disease varies substantially. We report a case of a female patient with TS presenting with pulmonary lymphangiomyomatosis and bilateral renal angiomyolipomas.

## Case presentation

A 31-year-old Caucasian Brazilian woman was admitted to the hospital with a 6-month history of chest pain and progressive dyspnea on exertion. She had angiofibromas on the malar regions of the face, which were present since her childhood. During her pregnancy, 11 years ago, she was diagnosed with polycystic kidney disease associated with tuberous sclerosis. She had repetitive urinary tract infections, which resulted in progressive loss of renal function. Both her grandfather and child had polycystic kidneys, and her son also presented seizures. The patient also had a history of hemorrhoidal disease causing intermittent bleeding.

On examination, the patient appeared pale, and her vital signs included a blood pressure of 120/80 mmHg and a heart rate of 110 bpm. Auscultation revealed the presence of fine crackles in both lungs, and a loud systolic heart murmur was heard on the precordium. Her abdomen was tense and painful to palpation, but there were no signs of peritoneal irritation. There was a palpable mass occupying the upper abdomen and both flanks; Traube's space was obliterated. Laboratory evaluation revealed a red blood cell (RBC) count of 2.81 × 10^6^/mm^3^, hemoglobin level of 9.0 g/dL, hematocrit of 27% and platelet count of 130 × 10^3^/mm^3^. Her WBC count was normal. Serum creatinine was 3.3 mg/dL; urea, 87 mg/dL; glucose, 85 mg/dL; sodium, 135 mEq/L; potassium, 4.2 mEq/L; uric acid 4.5 mg/dL; albumin, 2.8 g/dL; calcium, 7.9 mg/dL; phosphorus, 5.2 mg/dL; and magnesium, 2.4 mg/dL.

**Figure 1:**
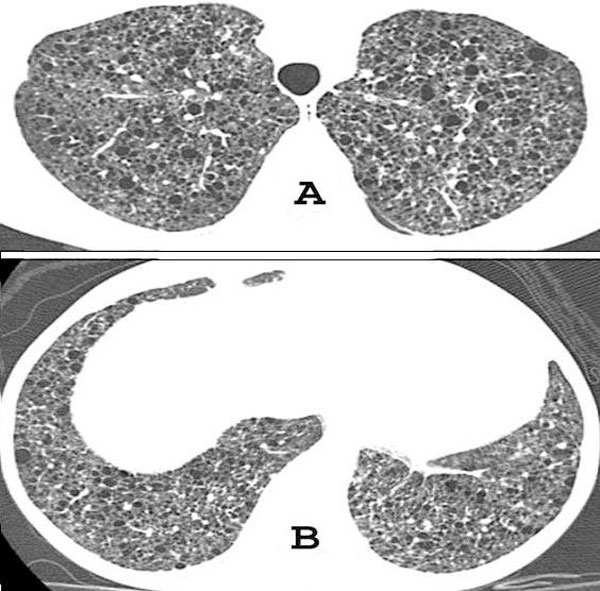
**High-resolution CT of the chest at the level of the upper **(A)** and lower lobes **(B)** shows well defined thin-walled cysts randomly scattered throughout both lungs**.

**Figure 2:**
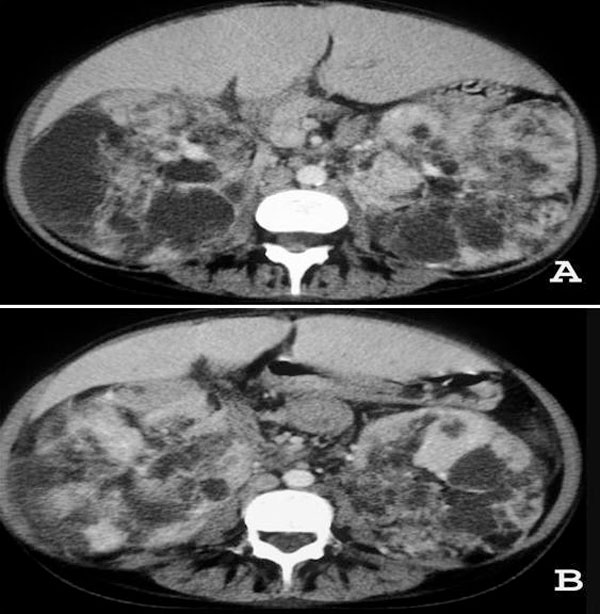
**CT of the upper abdominal region demonstrates bilateral giant renal masses consisting predominantly of fat tissue**. The density measurement of the hypodense content ranged from −15 to −148 Hounsfield units.

Chest computed tomography (CT) revealed cystic formations throughout the lungs, consistent with lymphangioleiomyomatosis (Figure 1), and the presence of a pericardial effusion. The echocardiogram showed a small pericardial effusion, and moderate left ventricular hypertrophy, and a normal systolic function. An abdominal CT scan demonstrated enlarged, heterogeneous kidneys, with multiple fat-density formations (negative densities, ranging from −15 to −148 Hounsfield units), which corresponded to angiomyolipomas (Figure 2). Pulmonary function tests showed a severe obstructive pulmonary disorder with reduced forced vital capacity, and a positive response to bronchodilator. She started treatment with medroxyprogesterone, and her respiratory status remained stable. An echocardiogram performed three years later revealed worsening of cardiac condition, enlargement of right cavities and left atrium, and thickening of the aortic valve leaflets; systolic function was preserved. Follow-up CT scans did not show significant changes.

## Discussion

The benign, non-invasive lesions of tuberous sclerosis can appear in any organ like the brain, heart, skin, eyes, kidney, lung, and liver. Therefore, TS has a wide clinical spectrum. The diagnosis of definitive TS is based on specific clinical features and requires the presence of two major criteria, or one major and two minor [3]. Pulmonary lymphangioleiomyomatosis, renal angiomyolipoma and facial angiofibroma are some of the major clinical features.

The most frequent cause of death in patients with TS is renal complication [3,5]. Multifocal, bilateral angiomyolipomas are found in about 70-90% of adult patients [3], and the prevalence increases with age, being less frequent in children [3,4]. These lesions are more often prevalent in women, suggesting a hormonal component to the tumor growth [6]. The angiomyolipomas are composed of varying amounts of mature adipose tissue, smooth muscle, and abnormal blood vessels [3,6]. The demonstration of intratumoral fat with negative attenuation values at CT is virtually pathognomonic of angiomyolipoma. Thin-section unenhanced CT is essential to visualize the fat content of angiomyolipomas [7]. Progressive enlargement of tumors and hemorrhage into the lesion can result in flank pain, a palpable tender mass and gross or microscopic hematuria, and interfere with renal function [6]. Tumors larger than 4 cm in diameter have a greater risk of spontaneous or traumatic rupture resulting in hemorrhagic complications [6], which is the most common cause of death in patients with TS [8]. Some patients with TS carry a contiguous germline deletion that affects both the *TSC2* gene and the adjacent gene, polycystic kidney disease type 1 (*PKD1*), resulting in a polycystic kidney phenotype that leads to early renal insufficiency [3,4]. In our patient, the family history indicates that she inherited a germline mutation in the *TSC2* gene. Renal cell carcinoma can occur in approximately 2-3% of adults with TS [3].

Pulmonary LAM is a rare progressive disease that predominantly affects women of childbearing age. Estrogen is thought to play a role in disease progression since it does not present prior to menarche and only rarely after menopause [9], and is exceptionally rare in men [1,3,8]. LAM probably affects 1-3% of patients with tuberous sclerosis [3,5]. Although some articles report the occurrence of LAM in 1 to 3% of the patients with TS [3,5], it seems that this incidence is much higher. Recent articles [10]-[12] report an incidence ranging from 26 to 34%. It is characterized by alveolar smooth-muscle proliferation leading to air trapping, pulmonary hemorrhage and lymphatic extravasation, and cystic destruction of the normal lung parenchyma [3]. Some of the manifestations are shortness of breath, coughing, chest pain, pneumothorax, chylous pleural effusions, hemoptysis, and eventually respiratory failure, but asymptomatic cases may occur [1,3,4]. Pulmonary function tests can show an obstructive or restrictive pattern [1]. Classical CT findings (diffuse, homogeneous, small thin-walled cysts) and compatible clinical history can be highly suggestive of LAM [5]. It is extremely difficult to treat, and the long-term prognosis is poor with the average duration of survival from the time of diagnosis near to 10 years [1]. Treatment consists of supportive management; hormonal therapy has been tried but without consistent success [1,9]. Sirolimus (rapamycin) is being explored as another potential treatment, but additional trials will be needed to assess efficacy and potential side effects [11,13].

Renal angiomyolipomas are present in 93% of patients with tuberous-sclerosis-associated pulmonary lymphangiomyomatosis [3]. It is important to recognize LAM before renal surgery for angiomyolipoma because of the risk of spontaneous pneumothorax or other perioperative pulmonary complication [5,8]. Pneumothoraces ultimately occur in approximately 60 to 70% of patients with LAM, and the rate of recurrence is >70%, the highest among all chronic lung diseases [11].

Finally, it is very important to understand that a patient with TS requires a multidisciplinary clinical staff to receive a complete evaluation of the multisystem complications. In patients with lymphangiomyomatosis, annual pulmonary-function testing may be useful to monitor lung function and provide a measure of disease progression [4]. The monitoring of angiomyolipomas growth, by ultrasonography, CT, or magnetic resonance, is an essential issue in the management of TS [4].

## Abbreviations

CT: computed tomography; LAM: lymphangiomyomatosis; TS: tuberous sclerosis.

## Consent

Written informed consent was obtained from the patient for publication of this case report and accompanying images. A copy of the written consent is available for review by the Editor-in-Chief of this journal. Funding was neither sought nor obtained.

## Competing interests

The authors declare that they have no competing interests.

## Authors' contributions

FV conceived the study. BL, JC, CA and IS performed the literature review. FV, EM, GZ, CM, AS and AV edit and coordinated the manuscript. All authors read and approved the final manuscript.
